# Transcriptomic analysis reveals and validates lipid raft–associated biomarkers and functional networks in Alzheimer’s disease

**DOI:** 10.1016/j.ibneur.2026.08.013

**Published:** 2026-08-14

**Authors:** Chun-Hsien Hsu, Amadou Wurry Jallow, Fatima Saleem, Thi Minh Chau Vu, Yung-Feng Lin

**Affiliations:** a Department of Family Medicine, Taipei City Hospital, Heping Fuyou Branch, Taipei 100, Taiwan; b Wanhua Outpatient Department, Taipei City Hospital, Heping Fuyou Branch, Taipei 100, Taiwan; c School of Medicine, College of Medicine, Fu Jen Catholic University, New Taipei 242, Taiwan; d Department of Exercise and Health Sciences, University of Taipei, Taipei 100, Taiwan; e Department of Mechanical Engineering, College of Engineering, National Yang Ming Chiao Tung University, Hsinchu 300, Taiwan; f Ph.D. Program in Medical Biotechnology, College of Medical Science and Technology, Taipei Medical University, New Taipei 235, Taiwan; g School of Medical Laboratory Science and Biotechnology, College of Medical Science and Technology, Taipei Medical University, New Taipei 235, Taiwan; h Department of Laboratory Medicine, Taipei Medical University Hospital, Taipei 110, Taiwan

**Keywords:** Alzheimer’s disease, Lipid rafts, Transcriptomics, Neuroinflammation, Synaptic dysfunction, Biomarkers

## Abstract

Alzheimer’s disease (AD) involves complex changes, including synaptic dysfunction, neuroinflammation, and metabolic impairment, yet the role of lipid raft–associated gene networks in these processes remains unclear. In this study, we performed an integrative transcriptomic analysis using the GSE5281 dataset and validated the findings in an independent cohort (GSE33000). By combining differential expression analysis with weighted gene co-expression network analysis, we identified 72 lipid raft–associated genes linked to mitochondrial, synaptic, and immune-related pathways. Using network analysis and machine learning approaches (LASSO and SHAP), we further identified eight key biomarkers (CBL, CD44, EZR, FAS, FYN, ITGB1, MAPK1, and TGFBR1) that showed strong diagnostic performance and consistent results across datasets. Functional analysis revealed increased inflammatory signaling, including pyroptosis and cytokine pathways, alongside reduced oxidative phosphorylation and synaptic activity. Interestingly, immune profiling showed only minor differences in immune cell infiltration, but clear activation of multiple immune pathways, such as Th2 and Treg signaling, CD8⁺ T-cell signatures, and IL-6/IL-10–mediated inflammation. These immune changes were strongly associated with lipid raft–related biomarkers. Overall, our findings suggest that lipid raft dysregulation may act as a key link between immune activation, mitochondrial dysfunction, and synaptic impairment in AD.

## Introduction

Alzheimer’s disease (AD) is the most common neurodegenerative disorder and the leading cause of dementia worldwide (2024 Alzheimer's disease facts and figures, 2024). Its prevalence is expected to rise substantially due to global population aging, posing a major public health and socioeconomic burden (Xiaopeng et al., 2025). Clinically, AD is characterized by progressive cognitive decline and loss of functional independence (Zheng and Wang, 2025), while neuropathologically it is defined by extracellular amyloid-β (Aβ) plaques and intracellular neurofibrillary tangles composed of hyperphosphorylated tau (Abdulkhaliq et al., 2026). Beyond these hallmark features, increasing evidence highlights the contribution of synaptic dysfunction, mitochondrial impairment, neuroinflammation, and lipid dysregulation to disease progression (Abdulkhaliq et al., 2026, Zhang et al., 2025), emphasizing the importance of membrane organization and lipid-dependent signaling in AD pathogenesis.

Within this broader framework, lipid rafts have emerged as critical membrane microdomains that integrate lipid metabolism with pathogenic signaling pathways (Viljetić et al., 2024, Simons and Ehehalt, 2002, Pantelopulos et al., 2024). These cholesterol- and sphingolipid-rich domains organize receptor clustering, signal transduction, and protein trafficking, which are essential for synaptic function (Simons and Ehehalt, 2002, Golub et al., 2004). Importantly, key components of amyloidogenic processing—including APP, β-secretase (BACE1), and the γ-secretase complex are localized within lipid rafts, where lipid composition modulates their interactions and activity (Hur, 2022). Alterations in lipid raft integrity can promote APP–BACE1 colocalization and enhance Aβ production, highlighting their active role in amyloidogenic signaling (Lee et al., 2025).

Neuroinflammation is a major driver of AD progression, characterized by chronic activation of microglia and astrocytes surrounding Aβ plaques (Deng et al., 2024, Han et al., 2025). These activated glial cells release pro-inflammatory cytokines and reactive oxygen species, exacerbating synaptic dysfunction and neuronal injury (Han et al., 2025). Although initially protective, prolonged inflammatory activation impairs Aβ clearance and promotes a self-sustaining cycle of neurodegeneration (Sobue et al., 2024). Notably, many immune receptors and signaling complexes are organized within lipid rafts, suggesting that membrane microdomain integrity critically regulates neuroinflammatory signaling in AD (Viljetić et al., 2024, Pantelopulos et al., 2024). However, the mechanisms linking lipid raft remodeling to glial activation remain incompletely understood (Li et al., 2025a).

Mitochondrial dysfunction is another central feature of AD, reflecting the high metabolic demands of neurons (Li et al., 2025b). Disruption of mitochondrial dynamics, including fusion, fission, and mitophagy, leads to accumulation of dysfunctional mitochondria, impaired ATP production, and increased oxidative stress (Grel et al., 2023, Ashleigh et al., 2023, Jurcău et al., 2022). Interactions with Aβ and tau further exacerbate mitochondrial impairment and disrupt energy supply to synapses (Monsalvo-Maraver et al., 2023, Berth and Lloyd, 2023). Alterations in mitochondrial biogenesis and quality control contribute to metabolic instability and neuronal vulnerability (Jurcău et al., 2022, Singh and Singh, 2025).

Importantly, neuroinflammation and mitochondrial dysfunction are functionally interconnected, forming a self-amplifying loop that drives synaptic impairment and neurodegeneration (Cicali et al., 2026, Kaur et al., 2019, Wang et al., 2025, Liu et al., 2025). Synaptic dysfunction, one of the earliest pathological features of AD, arises from this interplay and closely correlates with cognitive decline (Cicali et al., 2026). Inflammatory mediators impair mitochondrial function, while mitochondrial damage amplifies immune activation, accelerating neuronal injury (Kaur et al., 2019, Ebrahimi et al., 2025).

Advances in high-throughput omics technologies and bioinformatics have enabled large-scale investigation of AD-related molecular mechanisms (Cardillo et al., 2025, Mukherjee et al., 2025). Transcriptomic and network-based approaches, including weighted gene co-expression network analysis (WGCNA), facilitate identification of disease-associated gene modules linked to neuroinflammation, synaptic dysfunction, and metabolic impairment (Loi et al., 2025, Hamed et al., 2025). In addition, machine learning approaches have improved biomarker discovery and predictive modeling, supporting precision medicine strategies in AD (Olawade et al., 2025, Duygu, 2025).

Despite these advances, the molecular mechanisms linking lipid raft dysregulation to neuroinflammation, mitochondrial dysfunction, and synaptic impairment remain incompletely understood. In particular, how lipid raft–associated gene networks coordinate these interconnected pathological processes in AD has not been systematically explored. In this study, we performed an integrative transcriptomic and systems biology analysis to identify lipid raft–associated biomarkers and functional networks in AD. By combining differential expression analysis, network modeling, functional enrichment, and machine learning approaches, we aimed to elucidate the role of lipid raft–mediated signaling in AD pathogenesis and identify candidate biomarkers with diagnostic and therapeutic potential.

## Materials and methods

### Data source

**Fig. 1:**
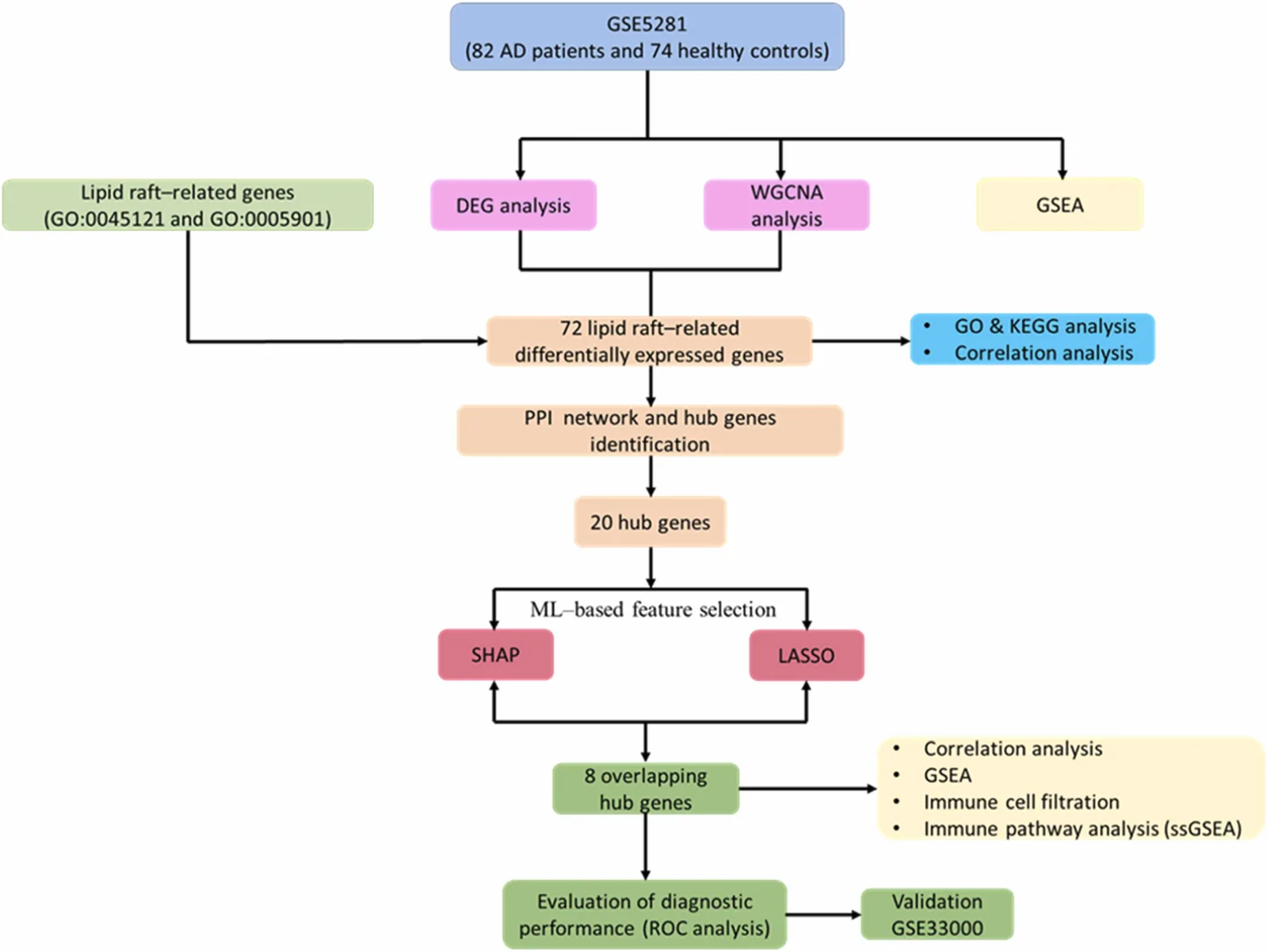
Workflow for the identification of lipid raft–associated biomarkers in AD. The GSE5281 dataset (82 AD and 74 control samples) was used as the training cohort. Lipid raft–related genes were obtained from Gene Ontology (GO:0045121 and GO:0005901) and integrated with differential expression analysis and weighted gene co-expression network analysis (WGCNA) to identify 72 lipid raft–associated differentially expressed genes (DEGs). Protein–protein interaction (PPI) network construction and hub gene analysis were subsequently performed to identify key gene modules. Feature selection using least absolute shrinkage and selection operator (LASSO) regression and SHapley Additive exPlanations (SHAP) analysis identified 8 candidate biomarkers. Functional enrichment analysis (GSEA), correlation analysis, immune cell infiltration analysis, and immune pathway analysis using single-sample gene set enrichment analysis (ssGSEA) were conducted to explore biological relevance. Finally, diagnostic performance was evaluated using receiver operating characteristic (ROC) analysis and validated in an independent dataset (GSE33000).

The workflow of the study is shown in Fig. 1. Gene expression datasets GSE5281 and GSE33000 were downloaded from the National Center for Biotechnology Information (NCBI) Gene Expression Omnibus (GEO) database (https://www.ncbi.nlm.nih.gov/geo/). AD cases in the GSE5281 and GSE33000 datasets were diagnosed based on post-mortem neuropathological evaluation, including the presence of amyloid-β plaques and neurofibrillary tangles, as described in the original studies (Liang et al., 2007, Narayanan et al., 2014, Liang et al., 2008). These criteria are consistent with established neuropathological diagnostic standards for AD (Braak and Braak, 1991).The GSE5281 dataset contains microarray gene expression profiles from brain tissues of 82 AD patients and 74 healthy controls, generated using the Affymetrix Human Genome U133 Plus 2.0 Array (GPL570) platform. The GSE33000 dataset includes expression profiles from brain samples of 310 AD patients and 157 healthy controls, also generated using the same microarray platform. Lipid raft–related genes were collected from the Gene Ontology (GO) database (https://www.informatics.jax.org/go/term/GO:0045121) and supplemented through literature review. Specifically, genes associated with the GO cellular component terms membrane raft (GO:0045121) and caveola (GO:0005901) were retrieved. After removing duplicate entries, a curated list of lipid raft–related genes was generated for subsequent analyses.

### Differential gene expression analysis

Differential gene expression analysis was performed to identify genes associated with AD using the GSE5281 dataset. Gene expression levels between AD and control samples were compared using the limma package (version 4.5.2) in R. P-values were adjusted for multiple testing using the Benjamini–Hochberg false discovery rate (FDR) method. Genes with an adjusted adjusted p-value < 0.05 and |log2 fold change| ≥ 0.5 were considered significantly differentially expressed. The results were visualized using a volcano plot (Tang et al., 2023), highlighting significantly upregulated and downregulated genes. The identified differentially expressed genes were subsequently used for downstream analyses, including WGCNA and pathway analysis.

### Weighted gene co-expression network analysis (WGCNA)

WGCNA was performed to identify gene modules associated with AD. The analysis was conducted using the WGCNA package in R. Gene expression data from the GSE5281 dataset were used as the training cohort for network construction. Prior to network analysis, genes with low variance across samples were removed to reduce noise and improve network stability. Hierarchical clustering of samples was first performed to detect potential outliers. Samples that did not cluster with the main dataset were excluded from further analysis. To construct a scale-free gene co-expression network, an appropriate soft-thresholding power (β) was determined using the scale-free topology criterion. The soft-thresholding power was selected based on the lowest power that achieved a scale-free topology fit index (R²) above the recommended threshold of 0.85. Using the selected soft threshold, an adjacency matrix was constructed to represent pairwise gene co-expression relationships. This adjacency matrix was then transformed into a topological overlap matrix (TOM) to measure network connectivity and reduce spurious associations. Genes were subsequently clustered using hierarchical clustering based on TOM dissimilarity. Gene modules were identified using the dynamic tree cutting algorithm, with each module representing a cluster of highly co-expressed genes. Modules with highly similar eigengenes were merged using a predefined similarity threshold. To investigate the biological relevance of the identified modules, module eigengenes MEs) were calculated and correlated with clinical traits, specifically AD status. Modules showing strong correlations with AD were considered candidate modules for further analysis.

### Identification of lipid raft–related differentially expressed genes

To identify lipid raft–associated genes involved in AD, the differentially expressed genes (DEGs) identified in the previous analysis were used for further investigation. In parallel, WGCNA was performed to identify gene modules significantly associated with AD. Modules showing significant correlations with AD status (r ≥ 0.2 or ≤ −0.2, p < 0.05), including both positively and negatively correlated modules, were considered AD-related modules. Genes belonging to these modules were extracted as candidate genes. A list of lipid raft–related genes, obtained as described in the previous section, was then incorporated into the analysis. The intersection among DEGs, genes from AD-associated WGCNA modules, and lipid raft–related genes was determined using a Venn diagram approach implemented in Venny 2.1 (https://www.bioinformatics.org/gvenn/index.htm). The overlapping genes were defined as candidate lipid raft–associated genes potentially involved in AD pathogenesis.

### Functional enrichment analysis

Functional enrichment analysis was performed to characterize the biological roles of lipid raft–related genes. Gene Ontology (GO) analysis identified enriched terms across biological process (BP), molecular function (MF), and cellular component (CC), while Kyoto Encyclopedia of Genes and Genomes (KEGG) analysis was used to identify associated signaling pathways (Sherman et al., 2022, Nguyen et al., 2024). In addition, Gene Set Enrichment Analysis (GSEA) was conducted using a ranked gene list based on log₂ fold change values from differential expression analysis. Genes were ordered from most upregulated to most downregulated between AD and control samples. GSEA was implemented using the clusterProfiler package in R (Wang et al., 2024), with gene sets obtained from the Gene Ontology Biological Process (GO-BP) collection via the msigdbr package. Enrichment was assessed using normalized enrichment scores (NES), with statistical significance determined by permutation testing and Benjamini–Hochberg false discovery rate (FDR) correction. Gene sets with FDR < 0.25 were considered significant. Pathway activity scores were calculated to examine relationships among key AD-related pathways, and correlation heatmaps were generated to visualize functional associations.

### Immune cell infiltration and immune pathway analysis

To comprehensively evaluate the immune microenvironment associated with Alzheimer’s disease (AD), both immune cell infiltration and immune-related pathway activity were analyzed using the GSE5281 expression dataset. The normalized gene expression matrix was log2-transformed (if necessary) and annotated to gene symbols prior to analysis. For immune cell infiltration analysis, immune cell–related signatures were defined using curated marker gene sets representing major immune cell populations, including CD8⁺ effector T cells, CD8⁺ memory T cells, natural killer (NK) cells, B cells, neutrophils, and dendritic cells. For each sample, immune cell scores were calculated as the average expression of corresponding marker genes and subsequently standardized using z-score normalization to enable comparability across cell types. Differences in immune cell signatures between AD and control groups were assessed using Welch’s *t*-test. To further characterize immune activity at the functional level, immune-related pathway signatures were quantified using single-sample gene set enrichment analysis (ssGSEA) implemented in the GSVA R package. Curated gene sets representing immune-related processes and cell-associated signatures were obtained from publicly available databases, including the Molecular Signatures Database (MSigDB) and previously published immune-related gene signatures (Xie et al., 2025, Li et al., 2023a). These gene sets encompassed pathways related to adaptive immunity (CD8⁺ T cells, Treg signaling, Th2 signaling), innate immune responses (neutrophil response, NK cell activation) (Sherman et al., 2022, Nguyen et al., 2024), inflammatory signaling (IL-6/IL-10–mediated pathways), and vaccine-related immune activation signatures. For each sample, ssGSEA was used to calculate enrichment scores representing the relative activity of each immune pathway, which were then normalized for downstream analyses. Differences in immune pathway activity between AD and control groups were assessed using the Wilcoxon rank-sum test. To investigate the relationship between immune features and lipid raft–associated processes, a lipid raft score was calculated as the mean expression of predefined lipid raft–related genes. In addition, a panel of lipid raft–associated biomarkers (CBL, CD44, EZR, FAS, FYN, ITGB1, MAPK1, and TGFBR1) was selected based on prior differential expression and machine learning analyses. Pearson correlation analysis was performed between (i) immune cell scores and lipid raft scores, and (ii) biomarker expression levels and immune pathway ssGSEA scores across all samples. P-values were adjusted for multiple comparisons using the Benjamini–Hochberg false discovery rate (FDR) method. Results were visualized using boxplots to compare immune cell and pathway activities between groups and heatmaps to illustrate correlations between lipid raft–associated features and immune-related signatures.

### Protein–protein interaction network construction and machine learning–based feature selection

To explore the functional interactions among lipid raft–associated genes, a protein–protein interaction (PPI) network was constructed using the STRING database (https://string-db.org) (Szklarczyk et al., 2019, Szklarczyk et al., 2024, Szklarczyk et al., 2023). The candidate lipid raft genes were uploaded to retrieve known and predicted interactions based on multiple evidence sources, including experimental data, curated databases, co-expression, and computational predictions. Interactions with a confidence score ≥ 0.4 were retained to ensure reliability. The resulting network was exported and visualized using Cytoscape (Hur, 2022). Network topology analysis was performed using the CytoHubba plugin to identify highly connected genes. Hub genes were ranked based on the Degree algorithm, which quantifies the number of direct interactions for each node. The top 20 genes with the highest degree values were defined as candidate hub genes for further analysis. To identify key predictive genes associated with AD, an integrated machine learning–based feature selection strategy was applied. First, a random forest model was constructed using the expression profiles of the selected hub genes, and SHAP (SHapley Additive exPlanations) analysis was performed to evaluate the contribution of each gene to model prediction. SHAP values were used to rank genes based on their importance in distinguishing AD from control samples. Second, Least Absolute Shrinkage and Selection Operator (LASSO) regression was applied to perform feature selection by identifying genes with non-zero coefficients, thereby reducing redundancy and minimizing overfitting. Finally, the intersection of genes identified by SHAP and LASSO was determined using Venn diagram analysis. These overlapping genes were defined as robust candidate biomarkers and were selected for downstream predictive modeling and validation.

### Receiver operating characteristic (ROC) analysis and external validation

To evaluate the predictive performance of individual hub genes, receiver operating characteristic (ROC) curve analysis was performed using both the training dataset (GSE5281) and an independent external validation dataset (GSE33000). In the training dataset, single-gene logistic regression models were constructed for each candidate biomarker. To ensure robustness and reduce overfitting, a stratified 5-fold cross-validation strategy was employed. Within each fold, gene expression values were standardized using z-score normalization based on the training subset, and the same transformation was applied to the corresponding test subset. Model training and prediction were performed within each fold, and ROC curves were generated accordingly. The mean ROC curve and the corresponding area under the curve (AUC) were calculated across all folds to provide a stable estimate of predictive performance for each gene. For external validation, the same logistic regression framework was applied to the independent GSE33000 dataset. Gene expression values were standardized prior to model fitting. To ensure consistency across datasets, gene symbols were harmonized and known aliases were standardized (VIL2 was mapped to EZR). ROC curves and AUC values were computed to assess the generalizability of each gene in an independent cohort. To facilitate direct comparison between training and validation results, identical visualization settings, including axis limits, color mapping, and plotting styles, were applied to all ROC curves.

### Visualization and statistical analysis

Visualization of gene expression and functional enrichment results was performed to characterize lipid raft–associated molecular alterations in AD. Differential expression results were displayed using volcano plots, in which genes were distributed based on log₂ fold change and statistical significance, with significantly upregulated and downregulated genes highlighted. Enrichment results were visualized using bubble plots, where point size represents −log10 adjusted P values and color encodes normalized enrichment scores (NES). Hierarchical clustering of enrichment profiles was applied to identify shared functional signatures across biomarkers. For expression analysis, biomarker expression values were extracted and standardized across samples using Z-score transformation. Heatmaps were generated to visualize expression patterns and differences between AD and control groups, with hierarchical clustering applied to genes and/or samples. Pairwise gene–gene relationships were quantified using Pearson correlation coefficients computed from normalized expression data. Correlation matrices were visualized as heatmaps, with color gradients indicating the strength and direction of associations. All statistical analyses were performed using R (v4.5.2) and Python (v3.9)**.** Key R packages included limma (v3.66.0) for differential expression analysis, clusterProfiler (v4.18.4) for gene set enrichment analysis, msigdbr (v25.1.1) for gene set retrieval, ggplot2 (v4.0.2) for visualization, and *pheatmap* (v1.0.13) for heatmap generation. Python-based analyses and visualization were conducted using pandas (v2.3.3)*,* NumPy (v2.3.5)*,* seaborn (v0.13.2*)*, and matplotlib (v3.10.6)*.* Multiple testing correction was performed using the Benjamini–Hochberg method, with a false discovery rate (FDR) < 0.05 considered statistically significant. All analyses were conducted using default parameters unless otherwise specified, and scripts were executed in a reproducible computational environment.

## Results

### Identification of lipid raft–associated differentially expressed genes and key co-expression modules in AD

**Fig. 2:**
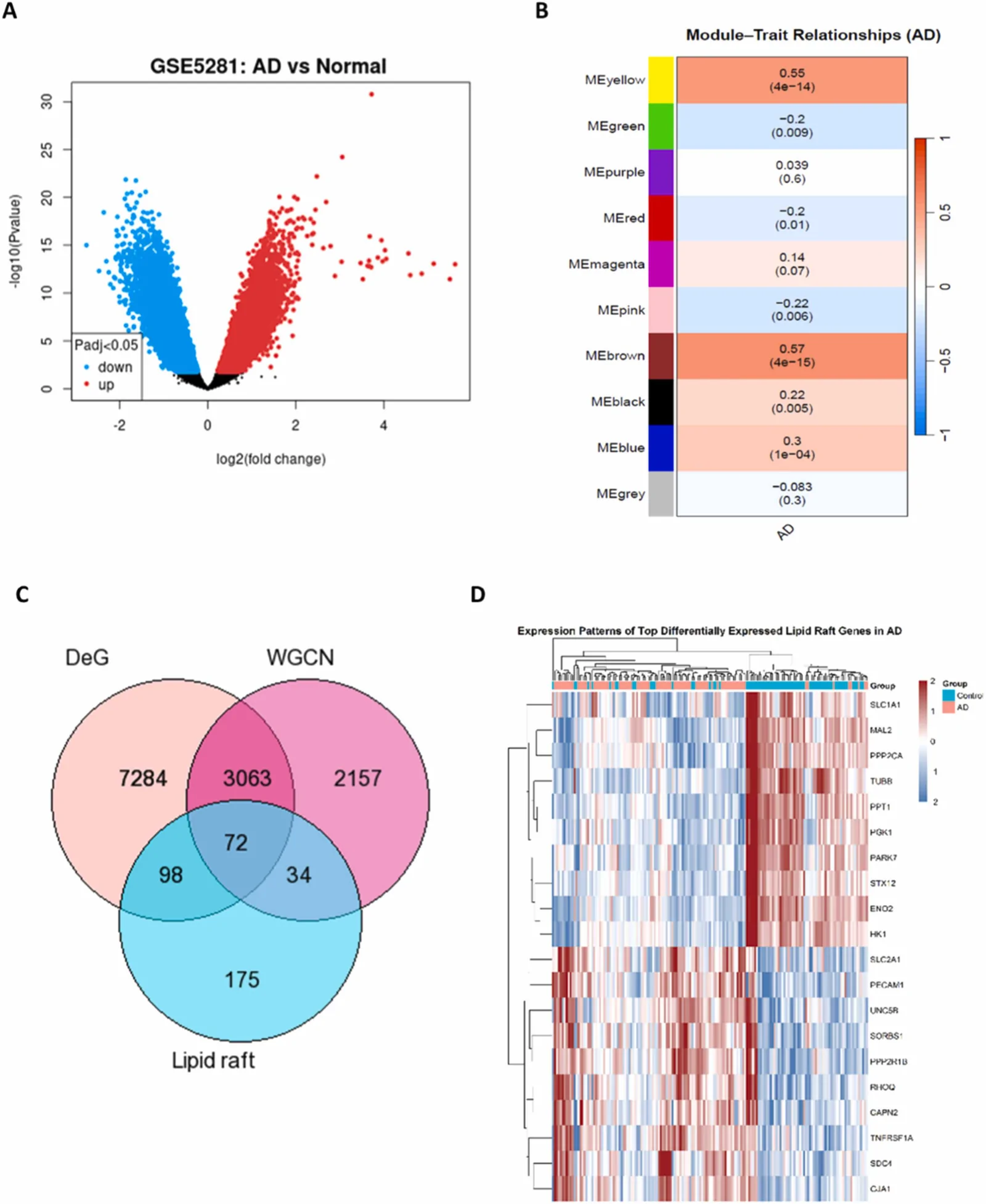
Identification of lipid raft–associated gene signatures in AD. (A) Volcano plot showing differentially expressed genes (DEGs) between AD and control samples in the GSE5281 dataset. Red and blue dots represent significantly upregulated and downregulated genes, respectively (adjusted p < 0.05). (B) Module–trait relationships identified by weighted gene co-expression network analysis (WGCNA). The heatmap displays correlations between gene modules and AD status, with correlation coefficients and corresponding p-values indicated. Modules showing strong positive or negative associations with AD were selected for further analysis. (C) Venn diagram illustrating the overlap among DEGs, WGCNA module genes, and lipid raft–related genes. A total of 72 overlapping genes were identified as lipid raft–associated DEGs. (D) Heatmap showing expression patterns of the top lipid raft–associated DEGs across AD and control samples. Distinct clustering patterns between groups indicate differential expression and underlying transcriptional heterogeneity.

Differential expression analysis of the GSE5281 cohort revealed widespread transcriptional dysregulation between AD and control samples (Fig. 2A). Quality assessment using hierarchical clustering confirmed sample consistency and excluded potential outliers (Supplementary figure 1 A). To further characterize coordinated transcriptional changes, weighted gene co-expression network analysis (WGCNA) was performed, identifying multiple gene modules associated with disease status (Fig. 2B; Supplementary figure 1B–C). Correlation analysis between module eigengenes and AD phenotype revealed that the brown (r **=** 0.57, p = 4 × 10⁻¹⁵) and yellow (r = 0.55, p = 4 × 10**⁻¹⁴)** modules exhibited the strongest positive associations with AD (Fig. 2B). In contrast, modules such as pink (r = −0.22, p = 0.006) and green (r = −0.20, p = 0.009) were negatively correlated with disease status, suggesting the presence of both activated and suppressed transcriptional programs in AD.

To identify lipid raft–associated genes relevant to AD, we integrated differential expression results, WGCNA-derived modules, and lipid raft–related Gene Ontology annotations. Intersection analysis revealed 72 overlapping genes, representing a core set of lipid raft–associated differentially expressed genes (Fig. 2C). These genes exhibited distinct expression patterns between AD and control samples, as demonstrated by hierarchical clustering (Fig. 2D).

Functional enrichment analysis showed that these lipid raft–associated genes were significantly enriched in biological processes related to membrane organization, cell adhesion, and signal transduction (Supplementary figure 2 A), as well as pathways including MAPK signaling and immune-related processes (Supplementary figure 2B).

### Lipid raft–associated transcriptional pathways link neuroinflammation, mitochondrial dysfunction, and synaptic impairment in AD

**Fig. 3:**
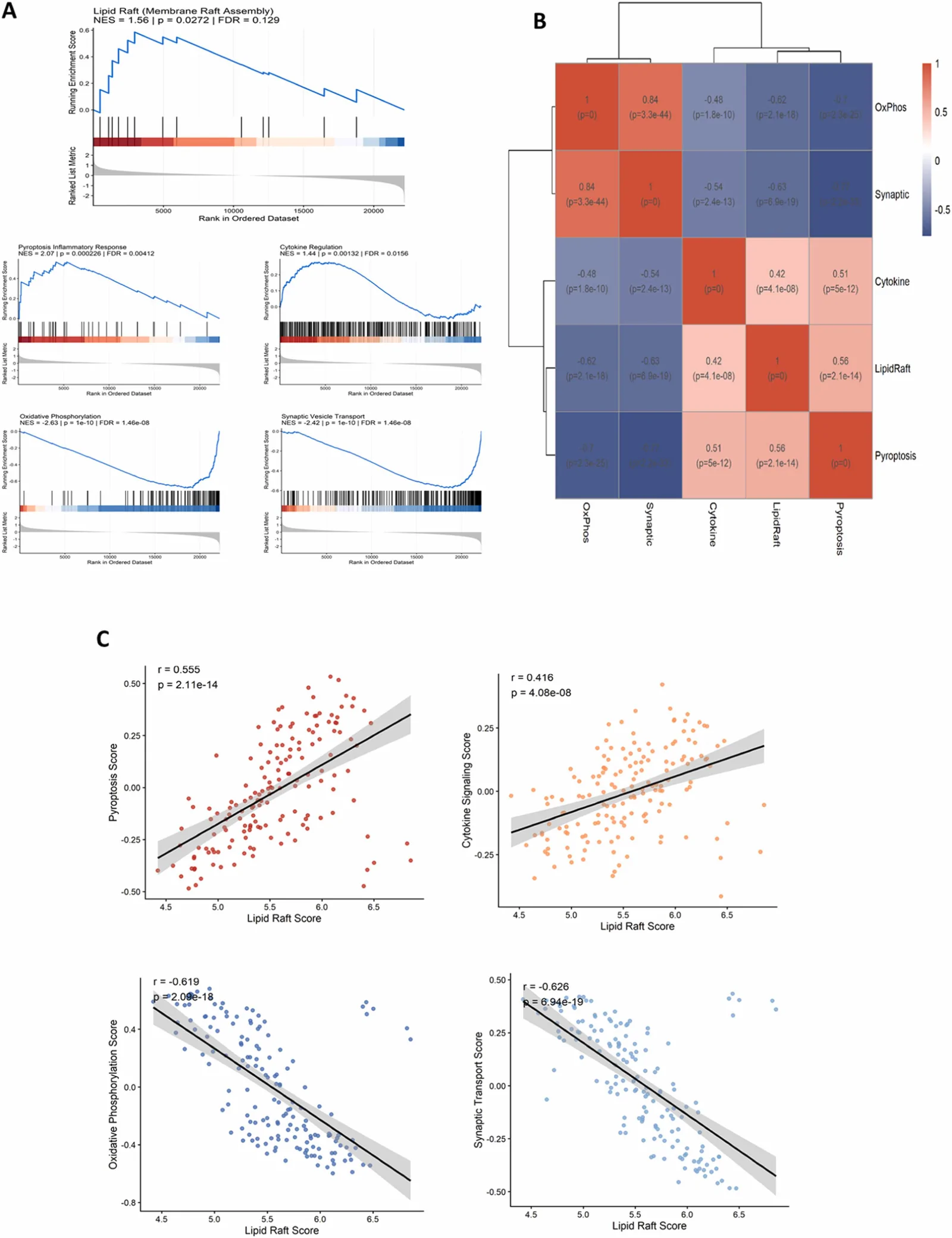
Lipid raft–associated transcriptional pathways and their relationships with mitochondrial dysfunction, synaptic impairment, and inflammatory activation in AD. (A) Gene set enrichment analysis (GSEA) plots showing enrichment of lipid raft–related processes (membrane raft assembly), inflammatory pathways (pyroptosis and cytokine signaling), and neuronal/metabolic pathways (oxidative phosphorylation and synaptic vesicle transport). Lipid raft, pyroptosis, and cytokine signaling pathways are positively enriched, whereas oxidative phosphorylation and synaptic vesicle transport are negatively enriched. (B) Correlation heatmap illustrating relationships among pathway activity scores. Lipid raft activity shows strong positive correlations with inflammatory pathways (pyroptosis and cytokine signaling) and negative correlations with oxidative phosphorylation and synaptic function. (C) Scatter plot analyses confirming these associations. Lipid raft scores are positively correlated with pyroptosis (r = 0.56, p < 0.001) and cytokine signaling (r = 0.42, p < 0.001), and negatively correlated with oxidative phosphorylation (r = −0.62, p < 0.001) and synaptic vesicle transport (r = −0.63, p < 0.001).

To investigate the biological processes associated with lipid raft dysregulation in AD, gene set enrichment analysis (GSEA) was performed using ranked gene expression profiles. Lipid raft–related processes, including membrane raft assembly (normalized enrichment score [NES] = 1.56, p = 0.0272, FDR = 0.129), were enriched, suggesting alterations in membrane microdomain organization in AD (Fig. 3A). Notably, inflammatory pathways were markedly activated. Pyroptosis (NES = 2.07, p = 2.26 × 10⁻⁴, FDR = 0.00412) and cytokine-related signaling (NES = 1.44, p = 0.00132, FDR = 0.0156) were significantly enriched, indicating enhanced inflammasome activity and dysregulated immune responses. In contrast, pathways associated with neuronal metabolism and synaptic function were significantly suppressed, including oxidative phosphorylation (NES = −2.63, p = 1 × 10⁻¹⁰, FDR = 1.46 × 10⁻⁸) and synaptic vesicle transport (NES = −2.43, p = 1 × 10⁻¹⁰, FDR = 1.46 × 10⁻⁸), reflecting impaired mitochondrial function and synaptic activity (Fig. 3A). To further characterize these relationships, correlation analyses were performed between lipid raft activity scores and pathway signatures. Inter-pathway correlation analysis demonstrated strong associations among lipid raft activity, pyroptosis, cytokine signaling, oxidative phosphorylation, and synaptic vesicle transport (Fig. 3B). Specifically, lipid raft activity showed significant positive correlations with inflammatory processes, including pyroptosis (r = 0.56) and cytokine signaling (r = 0.42), while exhibiting strong negative correlations with oxidative phosphorylation (r = −0.62) and synaptic vesicle transport (r = −0.63). These relationships were further validated by scatter plot analyses, which confirmed robust associations between lipid raft activity and both inflammatory activation and the suppression of oxidative phosphorylation and synaptic function (Fig. 3C).

### Lipid raft–associated gene networks are destabilized and rewired in AD

To investigate network-level alterations, differential correlation analysis was performed to compare gene–gene interactions between AD and control samples. The results revealed extensive remodeling of the lipid raft–associated gene network, characterized by widespread reductions in correlation strength alongside localized gains in coordinated expression (Supplementary Figure 3). Increased correlations were primarily observed among genes involved in membrane organization and immune signaling, including CD44, ITGB1, EZR, and TGFBR1/TGFBR2, suggesting enhanced coordination of lipid raft–associated and inflammatory processes. In contrast, reduced correlations involving key signaling and regulatory genes such as FYN, EGFR, and IKBKB indicate disrupted network connectivity and loss of coordinated regulation. Together, these data suggest that lipid raft dysregulation in AD is marked by global network destabilization accompanied by selective reinforcement of membrane- and immune-related interactions.

### Identification of hub genes and key biomarkers using machine learning approaches

**Fig. 4:**
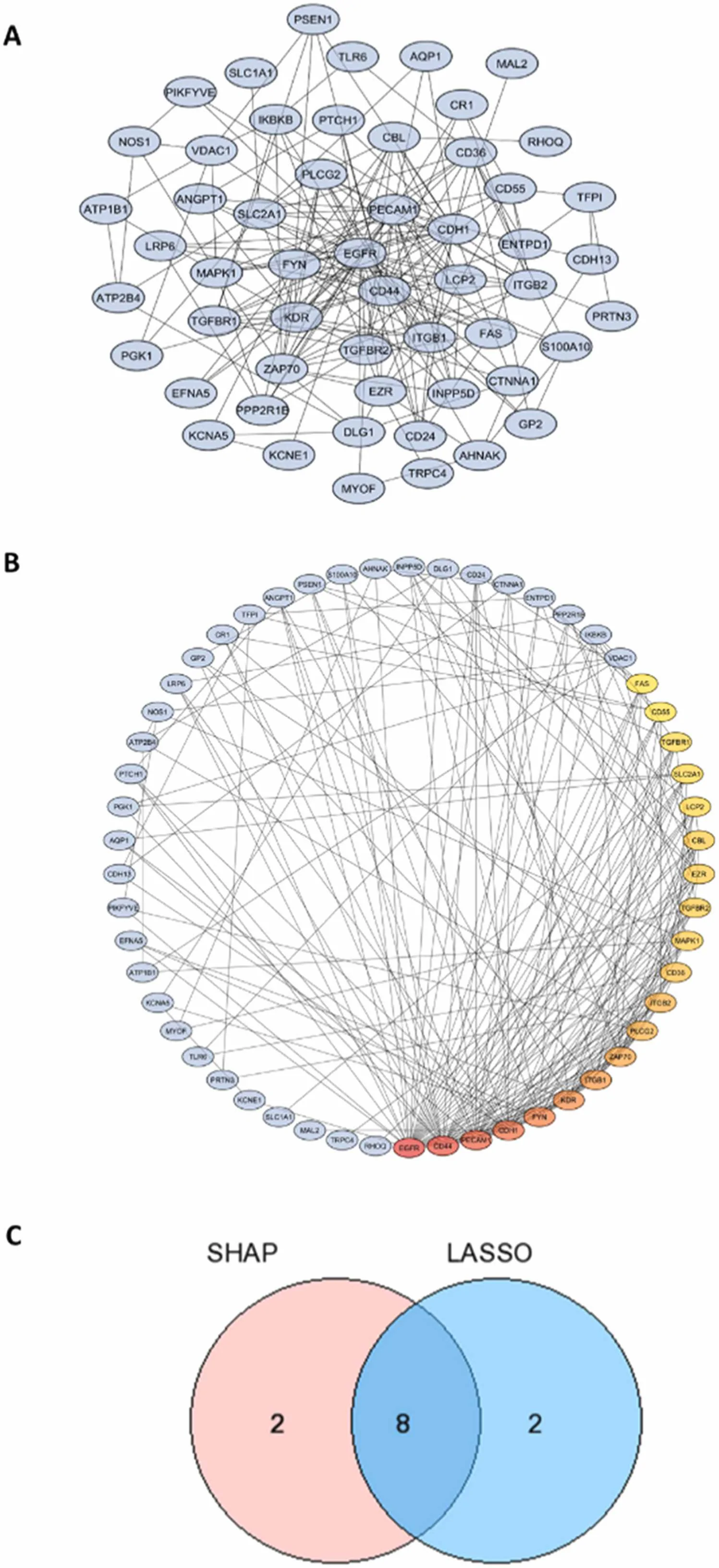
Identification of lipid raft–associated hub genes and key biomarkers in AD. (A) Protein–protein interaction (PPI) network of lipid raft–associated differentially expressed genes. (B) Network visualization highlighting hub gene distribution and connectivity. Nodes are colored according to their relative importance (centrality), with highly connected hub genes shown in warmer colors. (C) Venn diagram showing the overlap between feature selection methods. Eight genes were consistently identified by both LASSO and SHAP analyses.

To identify central regulators within the lipid raft–associated gene set, a protein–protein interaction (PPI) network was constructed, revealing highly interconnected nodes indicative of coordinated functional interactions (Fig. 4A). Network topology analysis using the Degree algorithm identified the top 20 highly connected genes as candidate hub genes (Fig. 4B). To further prioritize genes with predictive relevance for AD, machine learning–based feature selection was applied. SHAP analysis using a random forest model ranked genes according to their contribution to disease classification, with CBL and EZR emerging as the most influential features, along with CD44 and FYN (Supplementary figure 4A–B). In parallel, LASSO regression identified a subset of genes with non-zero coefficients, representing those with the strongest predictive power (Supplementary figure 4 C). Integration of SHAP and LASSO results revealed eight overlapping genes, including CBL, CD44, EZR, FAS, FYN, ITGB1, MAPK1, and TGFBR1, which were consistently selected across methods (Fig. 4C). These genes were considered robust lipid raft–associated biomarkers and were selected for downstream validation and diagnostic evaluation.

### Diagnostic performance of lipid raft–associated biomarkers in AD

**Fig. 5:**
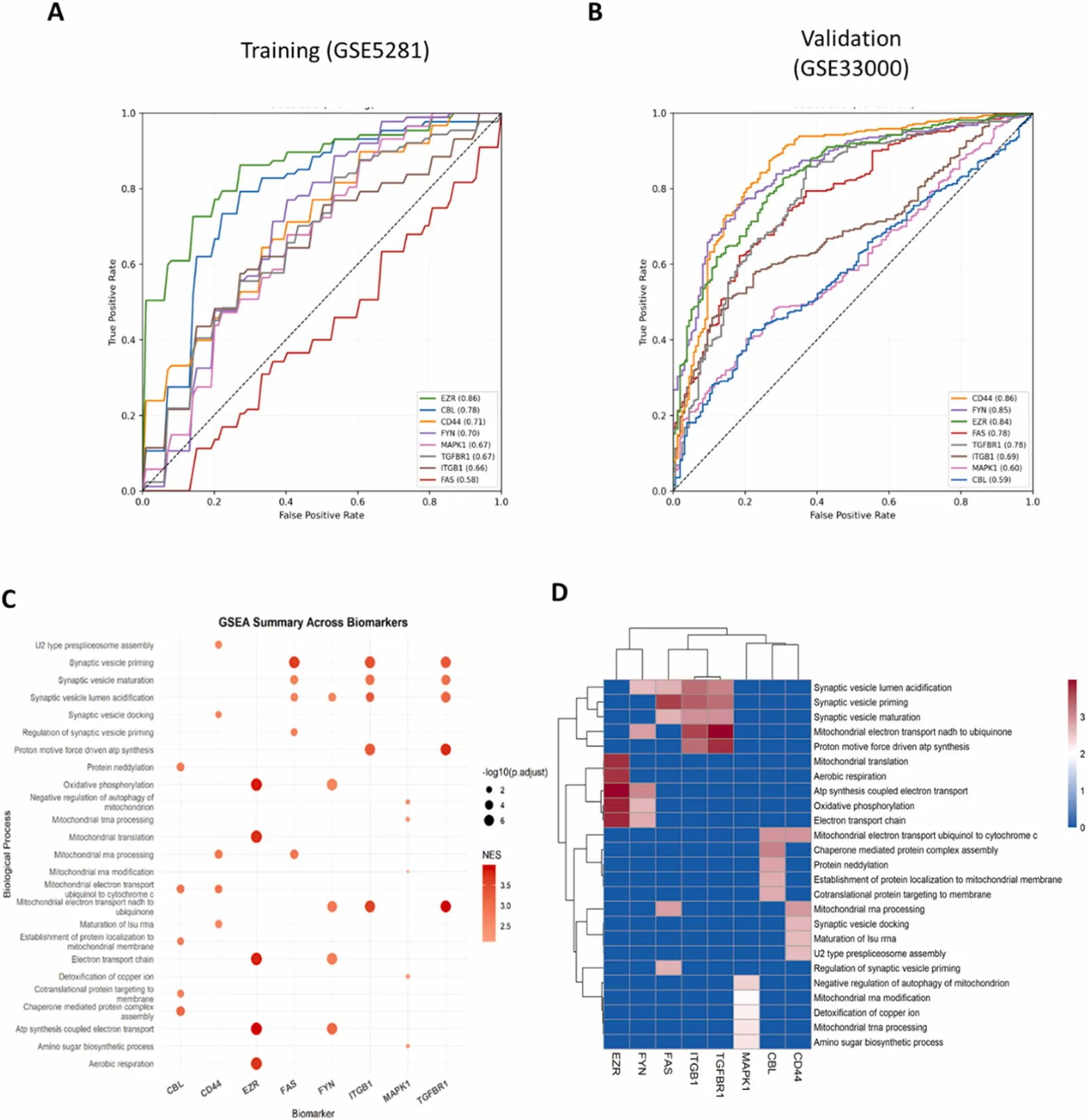
Diagnostic performance and functional characterization of lipid raft–associated biomarkers in AD. (A) Receiver operating characteristic (ROC) curves showing the diagnostic performance of individual lipid raft–associated biomarkers in the training cohort (GSE5281). (B) ROC curve analysis in the independent validation cohort (GSE33000), confirming the robustness and reproducibility of the selected biomarkers across datasets. (C) Gene set enrichment analysis (GSEA) summary showing biological processes associated with each biomarker. Dot size represents statistical significance, and color indicates the normalized enrichment score (NES). (D) Heatmap of pathway enrichment across biomarkers, illustrating distinct functional patterns.

To evaluate the diagnostic potential of the identified lipid raft–associated biomarkers, receiver operating characteristic (ROC) curve analysis was performed in both the training dataset (GSE5281) and an independent validation cohort (GSE33000). In the training dataset, several biomarkers demonstrated strong discriminatory ability between AD and control samples, with EZR showing the highest performance (AUC = 0.86), followed by CBL (AUC = 0.78), CD44 (AUC = 0.71), and FYN (AUC = 0.70) (Fig. 5A). Additional genes, including MAPK1, TGFBR1, and ITGB1, exhibited moderate predictive performance, while FAS showed relatively lower accuracy. In the validation dataset, the diagnostic performance of several biomarkers was largely preserved. CD44 (AUC = 0.86) and FYN (AUC = 0.85) exhibited the strongest classification performance, followed by EZR (AUC = 0.84) and TGFBR1 (AUC = 0.78) (Fig. 5B). Although some variability in performance was observed across datasets, the overall trends remained consistent.

### Expression patterns and inter-gene relationships of lipid raft–associated biomarkers in AD

Heatmap analysis of the selected biomarkers revealed distinct expression patterns between AD and control samples, with partial segregation of disease groups, reflecting underlying transcriptional heterogeneity (Supplementary Figure 5 A). Several genes, including CBL, CD44, EZR, FYN, and ITGB1, exhibited elevated expression in subsets of AD samples, suggesting their involvement in disease-associated molecular alterations. To further explore relationships among biomarkers, pairwise correlation analysis was performed. Strong positive correlations were observed between CBL and TGFBR1 (r = 0.68) and between CBL and FYN (r = 0.63), indicating coordinated regulation and potential involvement in shared signaling pathways (Supplementary Figure 5B). Additional moderate correlations among EZR, ITGB1, and FYN further support their roles in cytoskeletal organization and membrane-associated signaling. In contrast, weaker or negative correlations between MAPK1 and EZR suggest functional divergence within the lipid raft–associated network.

Differential expression analysis further confirmed that multiple biomarkers were significantly dysregulated in both the training (GSE5281) and validation (GSE33000) datasets (Supplementary Figure 5C–D). In the training cohort, CBL, CD44, EZR, FYN, and ITGB1 were significantly upregulated in AD, whereas MAPK1 was markedly downregulated. FAS and TGFBR1 did not show significant changes. In the validation cohort, most biomarkers, including CBL, CD44, EZR, FYN, and ITGB1, displayed consistent expression patterns, confirming their reproducibility across independent datasets. Compared with the training dataset, FAS and TGFBR1 showed increased expression in the validation cohort. Notably, MAPK1 exhibited an opposite trend, being downregulated in the training dataset but upregulated in the validation cohort, suggesting potential context-dependent regulation or dataset-specific variability. These analyses reveal that lipid raft–associated biomarkers exhibit coordinated expression patterns, interconnected regulatory relationships, and consistent dysregulation across cohorts, supporting their potential roles in AD pathogenesis and their utility as candidate diagnostic markers.

### Functional enrichment and pathway correlation of lipid raft–associated biomarkers in AD

To investigate the biological functions associated with the selected lipid raft–related biomarkers, GSEA was performed using ranked differential expression profiles. Across all biomarkers, enrichment analysis consistently highlighted pathways related to synaptic function and mitochondrial metabolism (Fig. 5C). Notably, genes such as FYN, ITGB1, and TGFBR1 were strongly associated with synaptic vesicle dynamics, including vesicle priming, docking, and maturation, reflecting their roles in synaptic signaling and structural organization. In parallel, MAPK1 and EZR were linked to pathways involved in signal transduction and cytoskeletal regulation, which are critical for maintaining synaptic integrity. Mitochondrial-related pathways, including oxidative phosphorylation and ATP synthesis, were prominently enriched and were associated with genes such as CBL, MAPK1, and FAS, suggesting a connection between lipid raft signaling and neuronal energy metabolism. In addition, enrichment of pathways related to autophagy and protein homeostasis was observed, with FAS and CD44 implicated in cellular stress responses and apoptotic signaling. To further characterize the relationships among these pathways, hierarchical clustering of enrichment scores revealed distinct but interconnected functional modules. Synaptic and mitochondrial pathways clustered together, indicating coordinated dysregulation across biomarkers (Fig. 5D). Collectively, these results suggest that lipid raft–associated biomarkers converge on shared biological processes involving synaptic regulation, mitochondrial function, and cellular stress responses, providing mechanistic insight into how lipid raft dysregulation may contribute to neuronal dysfunction in AD.

### Lipid raft–associated activity drives immune pathway activation despite limited changes in immune cell infiltration in AD

**Fig. 6:**
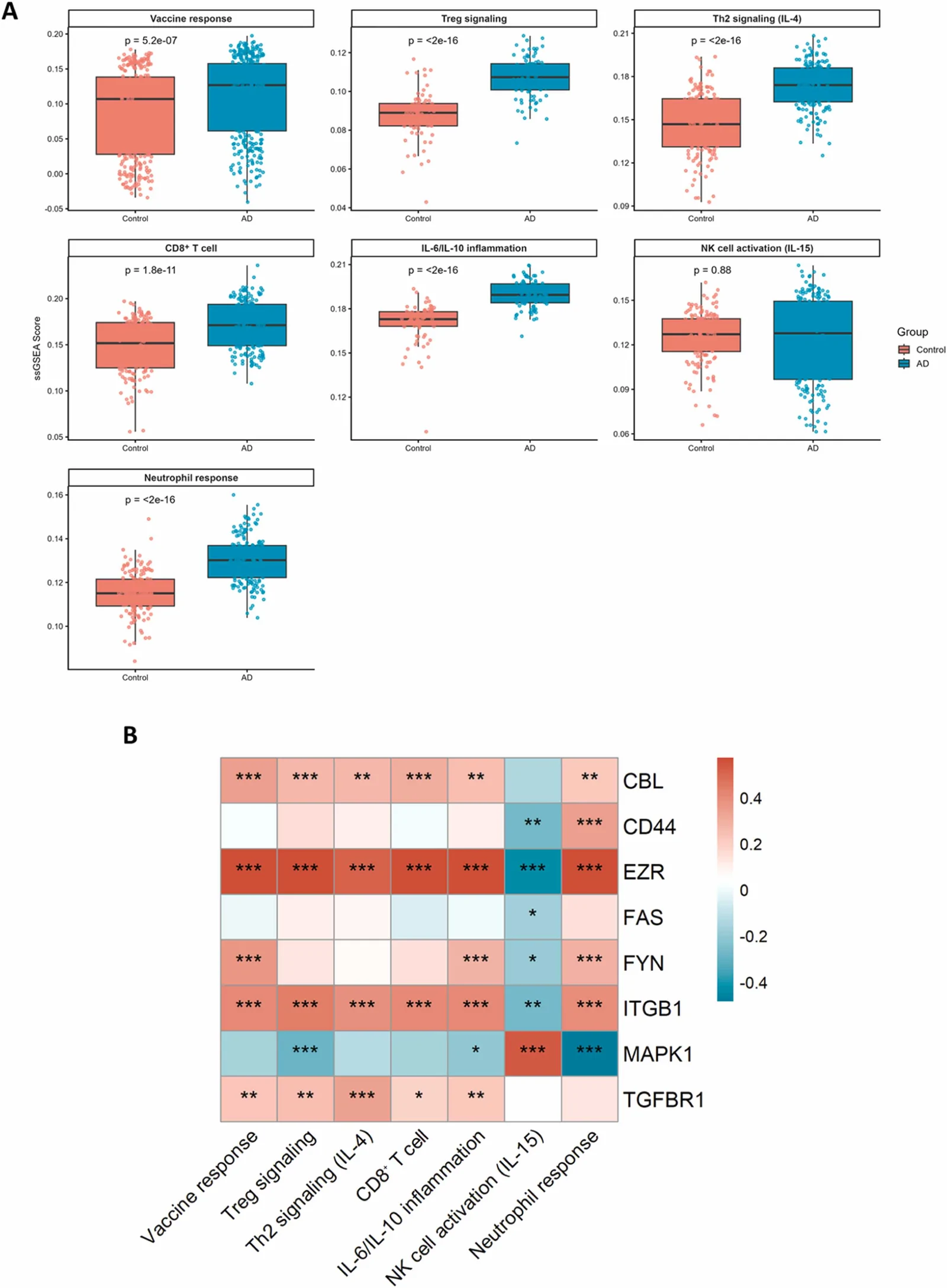
Immune pathway alterations and their association with lipid raft–related biomarkers in AD. (A) Comparison of immune pathway activity between control and AD samples based on ssGSEA scores. Boxplots represent the distribution of immune signatures, including vaccine response, Treg signaling, Th2 signaling (IL-4), CD8⁺ T cells, IL-6/IL-10 inflammation, NK cell activation (IL-15), and neutrophil response. Statistical significance was assessed using the Wilcoxon rank-sum test, with p-values displayed on each panel. (B) Correlation analysis between lipid raft–associated biomarkers (CBL, CD44, EZR, FAS, FYN, ITGB1, MAPK1, and TGFBR1) and immune pathway activity. Pearson correlation coefficients are represented by a color gradient (red, positive correlation; blue, negative correlation). Statistical significance is indicated as follows: *p < 0.05, **p < 0.01, and ***p < 0.001.

To evaluate the immune microenvironment in AD, immune cell signatures were first compared between AD and control samples. Among the analyzed cell types, only B cells showed a modest but statistically significant increase in AD compared to controls (p = 0.022) (Supplementary Figure 6 A). In contrast, CD8⁺ effector T cells, CD8⁺ memory T cells, natural killer (NK) cells, neutrophils, and dendritic cells did not exhibit significant differences between groups, although slight increasing trends were observed in AD samples. Despite these limited changes in immune cell abundance, analysis of immune-related pathways using ssGSEA revealed significant alterations at the functional level (Fig. 6A). Specifically, Th2 signaling (IL-4), Treg signaling, CD8⁺ T-cell signatures, IL-6/IL-10–mediated inflammation, neutrophil response, and vaccine-related immune responses were significantly elevated in AD compared to controls, whereas NK cell activation (IL-15) did not show a significant difference. These results indicate enhanced immune activity, particularly involving adaptive immune responses and inflammatory signaling, in AD.

To further investigate the relationship between immune activity and lipid raft–associated processes, correlation analyses were performed. Lipid raft activity showed strong positive correlations with multiple immune cell populations, including CD8⁺ memory T cells (r = 0.71, p < 0.001), B cells (r = 0.70, p < 0.001), CD8⁺ effector T cells (r = 0.62, p < 0.001), dendritic cells (r = 0.58, p < 0.001), NK cells (r = 0.54, p < 0.001), and neutrophils (r = 0.50, p < 0.001) (Supplementary Figure 6B). Consistently, correlation analysis between lipid raft–associated biomarkers and immune pathways further supported this association (Fig. 6B). Notably, CD44, EZR, and ITGB1 exhibited strong positive correlations with multiple immune signatures, including Treg signaling, Th2 signaling, CD8⁺ T cells, IL-6/IL-10 inflammation, and neutrophil response (all p < 0.01), suggesting central roles in linking lipid raft dynamics to immune activation. Similarly, CBL, FYN, FAS, and TGFBR1 showed moderate positive associations with immune pathways, indicating supportive roles in immune modulation. In contrast, MAPK1 displayed an opposing pattern, showing negative correlations with most immune signatures while maintaining a positive association with NK cell activation, suggesting a potential regulatory role within the lipid raft–immune signaling axis.

## Discussion

In this study, we performed an integrative transcriptomic analysis to identify lipid raft–associated gene signatures and biomarkers in Alzheimer’s disease (AD). By combining differential expression analysis, weighted gene co-expression network analysis (WGCNA), functional enrichment, immune profiling, and machine learning approaches, we identified a robust set of lipid raft–related genes that are strongly associated with AD pathology.

Lipid rafts are cholesterol- and sphingolipid-rich membrane microdomains that regulate signal transduction, synaptic organization, and protein trafficking. Increasing evidence indicates that lipid raft dysfunction contributes to AD pathogenesis by modulating amyloid precursor protein (APP) processing and synaptic signaling. For example, studies have shown that β-secretase (BACE1) activity and amyloid-β production are closely associated with lipid raft domains (Estrada et al., 2010, Vetrivel and Thinakaran, 2010). Consistent with this, our analysis identified 72 lipid raft–associated differentially expressed genes, enriched in membrane organization and signaling pathways, highlighting lipid rafts as a central molecular platform in AD. Beyond amyloid processing, lipid rafts serve as critical platforms for organizing signaling complexes that regulate neuronal function and survival. Disruption of lipid raft integrity has been shown to alter receptor localization and downstream signaling pathways, including those involving Src-family kinases such as FYN, which play key roles in synaptic plasticity and tau-related pathology. Evidence suggests that FYN-mediated signaling contributes to synaptic dysfunction and neurodegeneration in AD (Di Paolo and Kim, 2011, Nygaard et al., 2014, Scales et al., 2011). Lipid composition, particularly cholesterol homeostasis, is also essential for maintaining lipid raft stability. Alterations in brain cholesterol metabolism have been linked to changes in membrane microdomain organization and APP processing, thereby influencing amyloidogenic pathways (Di Paolo and Kim, 2011). In addition, lipid rafts are increasingly recognized as platforms for immune receptor signaling, including receptors involved in innate immune activation. These membrane domains facilitate the clustering of signaling molecules required for inflammatory responses, further linking lipid raft dysfunction to neuroinflammation in AD (Simons and Gerl, 2010).

One of the key findings of this study is the strong association between lipid raft signatures and mitochondrial dysfunction and synaptic deficits, two hallmarks of AD. We observed significant negative enrichment of oxidative phosphorylation and synaptic vesicle transport pathways, along with strong negative correlations between lipid raft activity and these processes. Mitochondrial dysfunction has long been implicated in AD, contributing to impaired ATP production, oxidative stress, and neuronal vulnerability (Swerdlow et al., 2014). Similarly, synaptic dysfunction is considered an early and critical event in AD progression, closely linked to cognitive decline (Selkoe, 2002). Our findings extend these observations by suggesting that lipid raft dysregulation may act upstream, linking membrane signaling alterations to both mitochondrial impairment and synaptic failure. Emerging evidence suggests that lipid rafts may play an upstream role in coordinating these processes by regulating membrane-associated signaling pathways that influence both mitochondrial function and synaptic integrity. Lipid rafts organize key signaling molecules involved in calcium homeostasis and receptor-mediated signaling, both of which are essential for mitochondrial activity and synaptic transmission (Simons and Gerl, 2010). Disruption of lipid raft composition can therefore impair neuronal energy metabolism and synaptic vesicle dynamics. Recent studies further support this connection. Alterations in membrane lipid composition and cholesterol metabolism have been shown to disrupt mitochondrial function and bioenergetics in AD models (Area-Gomez and Schon, 2017). In parallel, synaptic dysfunction has been linked to changes in membrane microdomain organization that affect receptor trafficking and neurotransmission efficiency (Sheng et al., 2012). More recent evidence also indicates that lipid–protein interactions at membrane domains influence mitochondrial dynamics and synaptic stability, reinforcing the role of membrane organization in maintaining neuronal homeostasis (Liu and Zhang, 2014, Zhang and Sima, 2024, Yin, 2023). Taken together, these findings support a model in which lipid raft dysregulation may act upstream, linking membrane signaling disturbances to both mitochondrial impairment and synaptic failure, thereby contributing to neurodegeneration in AD.

Neuroinflammation is a central feature of AD, and our study demonstrates that lipid raft–associated genes are closely linked to inflammatory and immune signaling pathways, including pyroptosis and cytokine-mediated responses. Pyroptosis, a form of inflammatory programmed cell death driven by inflammasome activation, has been increasingly implicated in neurodegenerative diseases (Heneka et al., 2018). Importantly, lipid rafts function as membrane platforms that facilitate the assembly and activation of immune receptors and inflammasome components, thereby amplifying inflammatory signaling cascades (Simons and Gerl, 2010). In the present study, immune profiling revealed that while overall immune cell infiltration exhibited minimal changes—except for a modest increase in B cells—immune-related pathways were significantly activated in AD. Specifically, Th2 signaling, Treg signaling, CD8⁺ T-cell–associated signatures, IL-6/IL-10–mediated inflammation, and neutrophil responses were elevated, indicating enhanced immune activity at the functional level rather than substantial alterations in immune cell abundance. Notably, lipid raft–associated biomarkers showed strong positive correlations with these immune pathways, suggesting that lipid raft dysregulation is more closely linked to functional immune activation than to changes in immune cell infiltration. These findings are consistent with previous studies demonstrating that peripheral immune components, particularly neutrophil-associated responses, contribute to AD pathology by promoting neuroinflammation, releasing reactive oxygen species, and disrupting the blood–brain barrier (Zenaro et al., 2015). More recent evidence further highlights the role of neutrophils and innate immunity in AD progression, showing that neutrophil extracellular traps (NETs) and associated inflammatory mediators exacerbate neuronal damage (Zenaro et al., 2015, Aries and Hensley-McBain, 2023). In addition, transcriptomic and single-cell analyses have revealed widespread dysregulation of immune responses in AD, characterized by activation of inflammatory and innate immune pathways alongside alterations in adaptive immune signaling (Leng and Edison, 2021). Importantly, our results extend these observations by demonstrating that lipid raft–associated genes are strongly correlated with both adaptive and innate immune pathways, suggesting a coordinated role in regulating immune signaling networks in AD. The differential behavior of MAPK1, which exhibited negative correlations with most immune pathways but a positive association with NK cell activity, further indicates that specific lipid raft–associated components may exert regulatory or balancing effects within the immune microenvironment. Together, these findings suggest that lipid raft dysregulation contributes to AD pathogenesis primarily through modulation of immune signaling pathways rather than large-scale changes in immune cell infiltration. By facilitating the organization and activation of immune-related signaling complexes, lipid rafts may promote sustained neuroinflammation and immune imbalance, thereby exacerbating disease progression.

Through integrated network and machine learning approaches, we identified eight key biomarkers (CBL, CD44, EZR, FAS, FYN, ITGB1, MAPK1, and TGFBR1) with strong diagnostic potential. Importantly, these genes are not isolated markers but are functionally interconnected and converge on key pathological processes in AD, including synaptic dysfunction, neuroinflammation, and cellular stress signaling. The eight lipid raft–associated biomarkers identified in this study represent diverse biological processes involved in AD pathogenesis. CBL (Casitas B-lineage lymphoma proto-oncogene) functions as an E3 ubiquitin ligase regulating receptor tyrosine kinase signaling and protein turnover. CD44 (cluster of differentiation 44) is a cell-surface adhesion receptor involved in immune activation and neuroinflammation. EZR (ezrin) is a membrane–cytoskeleton linker that regulates lipid raft organization and cell morphology. FAS (Fas cell surface death receptor, CD95) mediates apoptotic signaling and inflammatory responses. FYN (FYN proto-oncogene, Src family tyrosine kinase) regulates synaptic plasticity, NMDA receptor signaling, and tau phosphorylation. ITGB1 (integrin beta−1) participates in cell adhesion, extracellular matrix interactions, and synaptic stability. MAPK1 (mitogen-activated protein kinase 1; ERK2) is a key component of the ERK/MAPK signaling pathway regulating neuronal survival, synaptic plasticity, and inflammatory signaling. TGFBR1 (transforming growth factor-beta receptor 1) mediates TGF-β signaling involved in immune regulation, tissue remodeling, and neuroprotection. Among these, FYN, a Src-family tyrosine kinase, plays a central role in synaptic signaling and has been directly implicated in tau-mediated neurotoxicity. FYN interacts with tau and NMDA receptor complexes, promoting calcium influx and excitotoxic signaling, which contributes to synaptic dysfunction and neuronal loss in AD (Nygaard et al., 2014). This mechanism is further supported by studies showing that FYN-tau interactions enhance NMDA receptor–mediated excitotoxicity, a key driver of neurodegeneration (Liu et al., 2019). Cell adhesion–related molecules, including CD44 and ITGB1, are involved in extracellular matrix interactions and immune cell communication. CD44 has been associated with microglial activation and inflammatory signaling, while ITGB1 regulates cell–matrix adhesion and intracellular signaling pathways that influence neuronal survival and synaptic stability. These processes are closely linked to neuroinflammatory responses in AD, where glial activation and immune signaling play a central role in disease progression (Heneka et al., 2015). More recent studies further emphasize the importance of immune-related biomarkers and microglial dysfunction in AD pathogenesis (Zhang et al., 2021). MAPK1 represents a key node in intracellular signaling networks that regulate neuronal survival, plasticity, and stress responses. Aberrant activation of MAPK signaling pathways has been linked to inflammatory signaling and neuronal damage in AD, including pathways triggered by microglial activation and cytokine release (Bhaskar et al., 2014). TGFBR1, a central component of the TGF-β signaling pathway, plays a critical role in modulating neuroinflammation and glial activation. Recent studies indicate that immune-related signaling pathways, including those involving microglia and T cells, are dynamically regulated during AD progression, highlighting the importance of TGF-β–mediated signaling in maintaining immune homeostasis (McFarland and Chakrabarty, 2022).

In addition, CBL, an E3 ubiquitin ligase, is involved in receptor ubiquitination and degradation, suggesting a role in regulating receptor turnover and signal attenuation within membrane signaling domains (Tang et al., 2022, Ren et al., 2024). EZR links membrane proteins to the actin cytoskeleton, thereby maintaining membrane structure and facilitating signal transduction, and is also required for proper organization of membrane-associated signaling complexes, including those involved in apoptotic signaling (Fais et al., 2005, Li et al., 2025c, Kawaguchi and Asano, 2022, Pelaseyed and Bretscher, 2018). While, FAS, a key mediator of apoptotic signaling, contributes to neuronal loss through activation of programmed cell death pathways, particularly under conditions of chronic neuroinflammation (Li et al., 2023b). Overall, these biomarkers form a functionally interconnected network that integrates membrane signaling, cytoskeletal organization, immune activation, and neuronal stress responses. Their coordinated expression and strong inter-gene correlations observed in our study suggest that lipid raft–associated dysregulation operates through a multi-layered signaling network, rather than isolated pathways, ultimately driving synaptic dysfunction, neuroinflammation, and neurodegeneration in AD.

Importantly, several biomarkers demonstrated robust diagnostic performance across independent datasets, with area under the curve (AUC) values exceeding 0.80 for genes such as EZR, CD44, and FYN, indicating strong discriminatory power between AD and control samples. The reproducibility of these findings across independent cohorts highlights their potential utility as reliable transcriptomic biomarkers for AD diagnosis. Notably, transcriptomic biomarkers derived from disease-relevant pathways have been shown to improve diagnostic accuracy and reproducibility across cohorts (Aerqin et al., 2022). These biomarkers are functionally linked to key pathological processes, including membrane signaling, immune regulation, and synaptic function, suggesting that they capture core disease mechanisms rather than peripheral changes. This biological relevance enhances their translational value, as biomarkers reflecting underlying pathology are more likely to remain robust across disease stages and patient populations (Johnson et al., 2020).

From a clinical perspective, these genes may serve as components of multi-gene diagnostic panels, which have been increasingly recognized as more effective than single biomarkers for complex diseases such as AD (Hampel et al., 2023). Given that synaptic dysfunction and immune activation occur early in AD progression, lipid raft–associated biomarkers may be particularly valuable for identifying early or preclinical stages of disease (Jack et al., 2018).

Beyond diagnosis, these biomarkers also present potential therapeutic targets. For example, modulation of FYN signaling has been explored as a strategy to reduce synaptic toxicity and improve neuronal function (Nygaard et al., 2014). Similarly, targeting cell adhesion and integrin-related pathways, including CD44 and ITGB1, may influence neuroinflammatory responses and cellular signaling dynamics (Schwartz and Deczkowska, 2016). In addition, restoring lipid raft integrity through modulation of membrane lipid composition and cholesterol homeostasis has been proposed as a strategy to normalize neuronal signaling and reduce AD-related pathology (Di Paolo and Kim, 2011). Overall, these findings highlight the dual diagnostic and therapeutic relevance of lipid raft–associated biomarkers, supporting their potential application in precision medicine approach es for AD.

## Limitation

Despite the strengths of this integrative analysis, several limitations should be acknowledged. First, this study is based on publicly available transcriptomic datasets, and experimental validation at the protein and functional levels is required. Second, the cross-sectional design limits the ability to infer causal relationships. Third, the GSE5281 dataset includes samples from multiple brain regions derived from different individuals. Although this design provides broad coverage of AD-affected brain areas, it introduces potential heterogeneity that may influence gene expression patterns. In this study, efforts were made to account for this variability; however, residual region-specific effects cannot be completely excluded and may affect the robustness of the findings. Future studies should perform region-specific or stratified analyses to validate these results. Finally, bulk transcriptomic data do not capture cell-type–specific changes, which are particularly relevant in the heterogeneous brain microenvironment. Future studies should incorporate single-cell RNA sequencing, spatial transcriptomics, longitudinal datasets, and experimental models to further elucidate the mechanistic roles of lipid raft–associated genes in AD.

## Conclusion

This study identifies lipid raft–associated genes as key regulators linking neuroinflammation, mitochondrial dysfunction, and synaptic impairment in AD. Integrative transcriptomic and machine learning analyses revealed a set of interconnected biomarkers with consistent diagnostic performance across independent datasets. Our findings suggest that lipid raft dysregulation may act as an upstream driver of immune activation and neuronal dysfunction. These results provide new insight into AD pathogenesis and highlight lipid raft–associated pathways as promising targets for early diagnosis and therapeutic intervention.

## Author’s contributions

CHH, AWJ, and YFL conceived and designed the study. CHH, AWJ, FS, and TMCV performed analyses. CHH, AWJ and YFL interpreted data and wrote the manuscript.

## CRediT authorship contribution statement

**Yung-Feng Lin:** Writing – review & editing, Writing – original draft, Validation, Supervision, Project administration, Methodology, Investigation, Funding acquisition, Conceptualization. **Fatima Saleem:** Writing – original draft, Formal analysis, Data curation. **Thi Minh Chau Vu:** Writing – original draft, Data curation. **Chun-Hsien Hsu:** Writing – review & editing, Writing – original draft, Validation, Methodology, Investigation, Funding acquisition, Data curation, Conceptualization. **Amadou Wurry Jallow:** Writing – original draft, Visualization, Software, Methodology, Investigation, Formal analysis, Data curation, Conceptualization.

## Ethics declaration

Ethical approval and informed consent were not required for this study, as it was based on publicly available datasets.

## Funding

This study was sponsored by the 10.13039/501100015835Department of Health, Taipei City Government (11201–62–033) and the National Science and Technology Council (NSTC 111–2314-B−532–001), Taiwan (R.O.C.).

## Declaration of Competing Interest

The authors declare that they have no known competing financial interests or personal relationships that could have appeared to influence the work reported in this article.

## Data Availability

The data supporting the findings of this study are publicly available from the Gene Expression Omnibus (GEO) database (https://www.ncbi.nlm.nih.gov/geo). All software tools and analytical methods used are described within the manuscript.
