# Supplementary material for: Transcriptomic analysis reveals and validates lipid raft–associated biomarkers and functional networks in Alzheimer’s disease
Source: IBRO Neurosci Rep. 2026 Aug 14;21:625–38. doi: 10.1016/j.ibneur.2026.08.013 (PMC13521004; doi:10.1016/j.ibneur.2026.08.013)
Supplement: Supplementary file 1 [file mmc1.docx]

**Supplementary information**

**Supplementary figure 1.** Construction of weighted gene co-expression network and module detection. (A) Sample clustering dendrogram used to detect outliers based on gene expression profiles. The red horizontal line indicates the cut-off threshold, and all samples below the threshold were retained for subsequent analysis. (B) Gene clustering dendrogram with module identification using dynamic tree cutting. Different colors represent distinct gene co-expression modules, and the merged modules are shown below. (C) Scale-free topology analysis for selection of the soft-thresholding power. The left panel shows the scale-free topology fit index (R²) as a function of soft-threshold power, with the horizontal line indicating the target threshold (R² = 0.85). The right panel shows mean connectivity across different soft-thresholding powers. A suitable power was selected to balance scale-free topology and network connectivity.

**Supplementary figure 2.** Functional enrichment analysis of lipid raft–associated genes in AD. (A) GO enrichment analysis showing significantly enriched terms across three categories: biological process (BP), cellular component (CC), and molecular function (MF). (B) KEGG pathway enrichment analysis of lipid raft–associated genes. The x-axis represents −log10(P-value). Dot size indicates gene count; color represents gene ratio.

**Supplementary figure 3**. Differential correlation network of lipid raft genes in AD. Heatmap shows changes in pairwise gene correlations (Pearson r, AD − Control), highlighting widespread network rewiring with both strengthened and weakened interactions.

**Supplementary figure 4.** Identification of key lipid raft–associated biomarkers using machine learning. (A) SHAP feature importance plot showing the average contribution of each gene to the predictive model. Genes with higher mean absolute SHAP values have greater influence on model output. (B) SHAP summary (beeswarm) plot illustrating the distribution of feature effects across samples. Each point represents an individual sample, with color indicating relative gene expression levels. Positive SHAP values indicate increased contribution to AD prediction, whereas negative values indicate reduced contribution. (C) LASSO regression analysis showing the coefficients of selected genes. Genes with larger absolute coefficients contribute more strongly to the model, with both positive and negative coefficients indicating direction of association.

**Supplementary figure 5.** Expression patterns and correlation of lipid raft–associated biomarkers in AD. (A) Heatmap showing Z-score–normalized expression levels of selected biomarkers (CBL, CD44, EZR, FAS, FYN, ITGB1, MAPK1, and TGFBR1) across samples in the training dataset (GSE5281). Samples are annotated by condition (control and Alzheimer’s disease, AD), and hierarchical clustering was performed to identify expression patterns. (B) Correlation heatmap displaying pairwise Pearson correlation coefficients among the selected biomarkers. Color intensity represents the strength and direction of the correlation (red, positive; blue, negative), indicating coordinated expression relationships between genes. (C) Violin plots showing the distribution of biomarker expression levels between control and AD samples in the training dataset (GSE5281). (D) Validation of expression patterns in an independent dataset (GSE33000). Violin plots demonstrate consistent differential expression across cohorts. Statistical significance was assessed using Wilcoxon rank-sum test, with significance levels indicated as: ns, not significant; * p < 0.05; **p < 0.01; ***p < 0.001.

**Supplementary figure 6.** Immune cell signatures and their association with lipid raft activity in AD. (A) Comparison of immune cell scores between control and AD samples based on marker gene–derived signatures. Boxplots represent the distribution of scores for CD8⁺ effector T cells, CD8⁺ memory T cells, natural killer (NK) cells, B cells, neutrophils, and dendritic cells. Statistical significance was assessed using Welch’s t-test. (B) Correlation between lipid raft activity and immune cell signatures across all samples. Heatmap displays Pearson correlation coefficients (r), with color gradients indicating the strength of association. Significance levels are indicated as follows: p < 0.05, ***p < 0.001.
